# Pazopanib for metastatic pulmonary epithelioid hemangioendothelioma—a suitable treatment option: case report and review of anti-angiogenic treatment options

**DOI:** 10.1186/s12885-015-1395-6

**Published:** 2015-05-13

**Authors:** Valeriya Semenisty, Inna Naroditsky, Zohar Keidar, Gil Bar-Sela

**Affiliations:** 1 Integrated Oncology and Palliative Care Unit, Rambam Health Care Campus and Technion-Israel Institute of Technology, POB 9602, Haifa, 31096 Israel; 2 Institute of Pathology, Rambam Health Care Campus and Technion-Israel Institute of Technology, Haifa, Israel; 3 Department of Nuclear Medicine, Rambam Health Care Campus and Technion-Israel Institute of Technology, Haifa, Israel

**Keywords:** Epithelioid hemangioendothelioma, Pulmonary, Pazopanib, VEGFR

## Abstract

**Background:**

Epithelioid hemangioendothelioma is a rare vascular tumor of borderline or low-grade malignancy. The lungs and liver are the two common primary organs affected. Metastatic disease was reported in more than 100 cases in the literature. However, no firm conclusions can be determined for recommended treatment options.

**Case presentation:**

The current case presents a patient with metastatic pulmonary epithelioid hemangioendothelioma to the cervical and mediastinal lymph nodes, lungs and liver that has been treated with pazopanib for more than two years with PET avid complete metabolic response in the mediastinum and lungs, and long-lasting stable disease. Target therapies that block VEGFR have a logical base in this rare malignancy.

**Conclusions:**

The current case is the first to report objective, long-lasting response to pazopanib.

## Background

Pulmonary epithelioid hemangioendothelioma (PEH) was first described by Dail *et al.* in 1983, who called it an intravascular bronchioloalveolar tumor [1]. Development of immunohistochemical techniques confirmed its endothelial lineage, and Wiess *et al.* subsequently suggested the current name, “epithelioid hemangioendothelioma” [2]. Immunohistochemistry for PEH showed diffuse cytoplasmic staining of the malignant cells, with some or all of the vascular-endothelial markers (CD31, CD34 and factor VIII) [3].

Epithelioid hemangioendothelioma (EHE) is a rare vascular tumor of borderline or low-grade malignancy. The lungs and liver are the two common organs for primary EHE, but it can spread through the bloodstream to other sites, such as bone and soft tissue. According to a literature review, nearly 100 cases have been described, mainly discussing a differential diagnosis [4]. The treatment options in metastatic disease are not well established. The current case presents a patient with metastatic PEH that was treated with pazopanib as first line of treatment.

## Case presentation

In December 2011, a 62-year old woman was referred to our Emergency Department with a history of progressive chest pain in the preceding 3 months. She had no prior medical history, was a non-smoker, and denied any history of cardiovascular diseases. CT scan revealed multiple nodules in both lungs up to 6 mm in diameter, multiple cervical lymph nodes up to 10 mm, and unclear lesions in the liver.

**Fig. 1:**
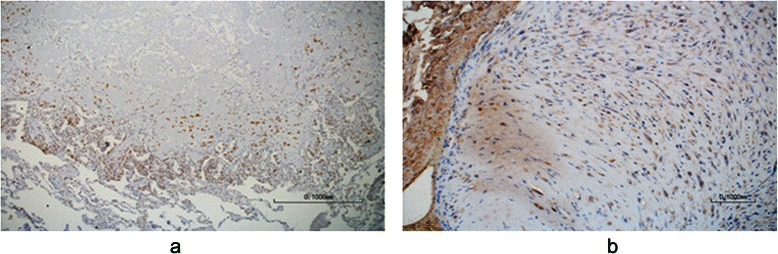
Immunohistochemical staining. **a** Immunostain for ERG, showing strong nuclear stain of the tumor cells. Original magnification × 100. **b** Immunostain for VEGF-R1, also designated Fms-like tyrosine kinase 1 (Flt-1), shows strong cytoplasmic staining of tumor cells. Original magnification × 200

For pathological diagnosis, the patient underwent thoracoscopic surgery with wedge resection of two lesions from the right lung. Immunohistochemical (IHC) stains demonstrated positive staining for endothelial markers CD31, CD34, FLI-1, and ERG, representing epithelioid hemangioendothelioma. The stain for ERG is shown in Fig. 1a. IHC was performed also for vascular endothelial growth factor receptor 1 (VEGFR1), and was found to be strongly positive (Fig. 1b).

**Fig. 2:**
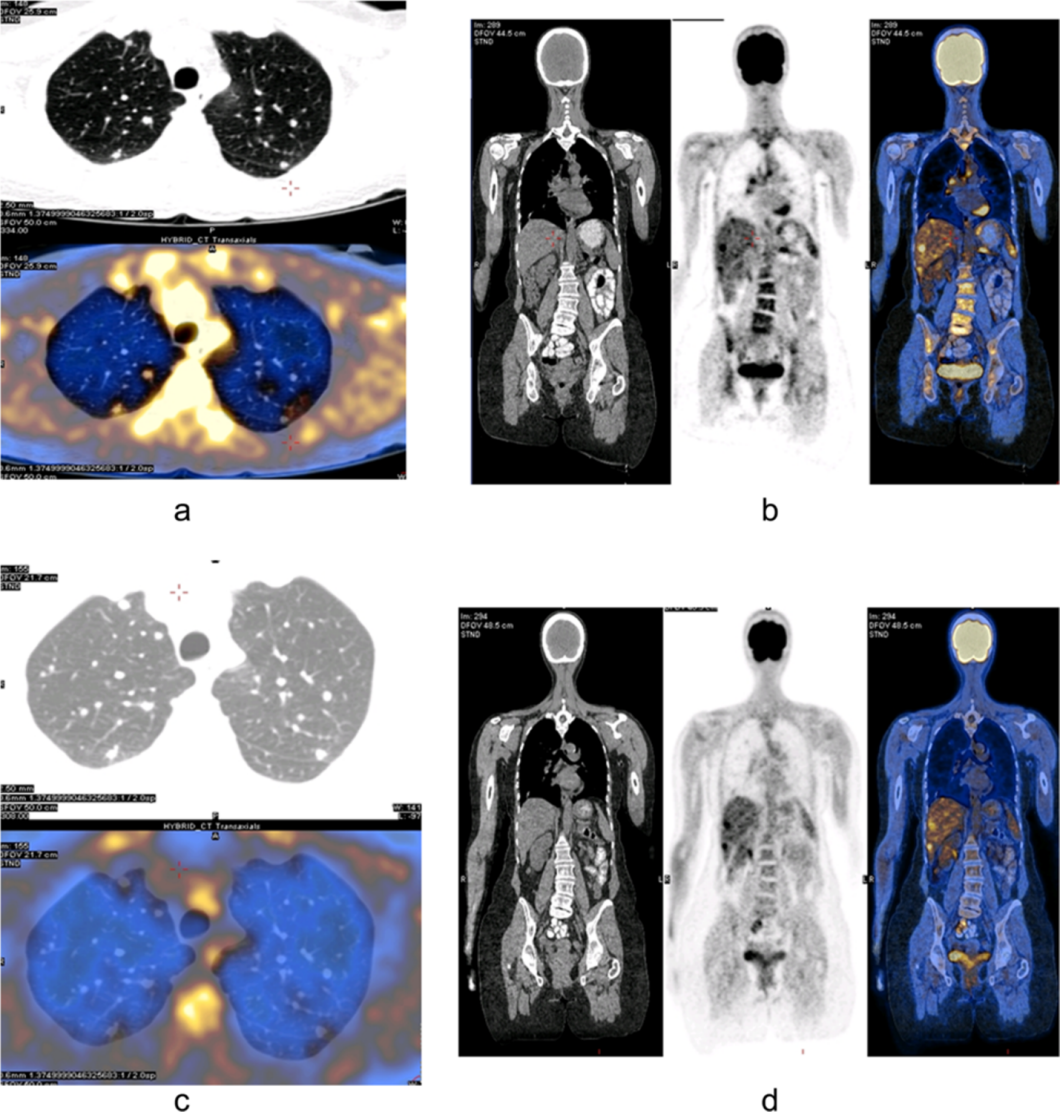
18F-FDG PET-CT. **a** PET-CT (selected axial slice) performed at staging, demonstrates pathological FDG foci in a few lung nodules. **b** PET-CT (selected coronal slice) performed at staging, demonstrates pathological FDG foci in mediastinal lymph nodes and the liver. Additional findings were demonstrated in a few cervical lymph nodes (not shown). **c** PET-CT (selected axial slice) performed after treatment, demonstrates a few lung nodules with no FDG uptake. **d** PET-CT (selected coronal slice) performed after treatment, demonstrates pathological FDG foci in the liver. No mediastinal findings are shown

In March 2012, before treatment was started, for final evaluation of unclear liver lesions, 18F-FDG PET-CT was performed and showed increased pathological uptake of 18F-FDG in the pulmonary nodules, cervical and mediastinal lymph nodes, and the liver (Fig. 2a, b).

Following the advanced disease shown by the PET-CT and the patient’s dyspnea, treatment with pazopanib in the standard dose of 800 mg orally once daily was started in April 2012. The treatment was given for more than 2 years without any side effects, except grade I fatigue. Other treatment options, such as interferon-alpha or chemotherapy, were discussed with the patient before treatment but were postponed by the patient due to concerns of possible side effects.

Since the disease had been initially demonstrated on PET scan, FDG-PET-CT was performed again in February 2013 and demonstrated disappearance of the pathological uptake in the mediastinal lymph nodes and in the lung lesions, with reduced metabolic response in the liver (Fig. 2c, d). The last FDG-PET-CT in June 2014 showed stable disease, without changes compared to February 2013.

## Discussion

According to a literature review, only 108 cases of this rare tumor involving the lungs have been published. The largest series of PEH published in 2006 contained 93 cases. The authors found an average age of 40.1 ± 17.3 years, with a female predominance of 73 %. Almost half the patients (49.5 %) were asymptomatic at diagnosis. Reported symptoms were dyspnea and cough (18.3 % each), chest pain (16 %), hemoptysis and weight loss (6.5 % each) [4].

Epithelioid hemangioendothelioma can be primary in the lung or pleura, or it may arise in liver, soft tissue or bone. The prognosis is very unpredictable, with life expectancy ranging from 1 to 15 years [5].

The poor prognostic factors of PEH include the presence of respiratory symptoms or pleural effusion at diagnosis, extensive intravascular, endobronchial or interstitial tumor spreading, hepatic metastases, peripheral lymphadenopathy, or the presence of spindle cells in the tumor [2]. However, the worst prognosis was for patients with pleural effusion or hemoptysis, with a median survival of less than 1 year [4]. The current patient had several poor prognostic factors (respiratory symptoms, hepatic metastases, and peripheral lymphadenopathy).

There is no established standard treatment for PEH, due to the rarity of the disease. Surgical resection should be performed if possible. In asymptomatic patients with diffuse lesions, watchful waiting is an acceptable option [1, 6]. Radiotherapy is not effective in certain patients due to the slow growth of the tumor cells, and chemotherapy appears to have little effect [7–9]. A few cases reported response or stable disease following immunotherapy treatment with interferon alpha [10–14].

Although its etiology remains unknown, immunohistochemical and electron microscopy studies have revealed that PEH is of endothelial origin [3]. Lymphatic dissemination is extremely rare, thus supporting the endothelial origin of the tumor. Vascular endothelial growth factor (VEGF) and the VEGF receptor were found on PEH tumor cells [15, 16], suggesting that VEGF inhibitors may be a potential treatment for PEH. In a review published a few years ago, anti-angiogenesis agents in angiosarcoma and EHE are discussed but, except for specific activating mutations in VEGFR2, which may be effectively targeted by VEGFR TKIs in some angiosarcomas, the biological mechanisms underlying the activity of these agents in angiosarcoma and EHE are poorly understood [17]. However two small phase II studies were performed with anti-angiogenic drugs in EHE. In a study by Agulnik *et al.*, testing the effect of bevacizumab alone in angiosarcoma and EHE, seven patients with EHE were included; two had partial response (PR) and four had stable disease (SD) [18]. In a sub-group report of 15 patients with EHE who were included in the phase II study of the French Sarcoma group testing the effect of sorafenib in sarcoma patients, only two had PR and five had SD [19].

Pazopanib is a second-generation tyrosine kinase inhibitor with highly selective activity against VEGFR, PDGFR, and c-KIT, which has demonstrated significant clinical benefit in a variety of malignancies, especially for the treatment of metastatic renal cell carcinoma [20]. The PALETTE (Pazopanib Explored In Soft Tissue Sarcoma) study was the first randomized phase III trial demonstrating the efficacy of this anti-angiogenic agent in pretreated soft tissue sarcoma (STS) patients, and 10 % of the patients in the pazopanib group had low-grade sarcomas [21].

In the current case, the patient is still on treatment with pazopanib, with partial response after a few months and prolonged stable disease for up to 24 months based on follow-up with a CT-PET-FDG scan. Considering that chemotherapy is generally ineffective in epithelioid hemangioendothelioma, angiogenesis inhibition is a reasonable approach to manage patients with metastatic EHE.

In a literature review for PEH cases and different target anti-angiogenetic medication, only eight patients who received chemotherapy and bevacizumab were found [6, 9, 15, 16, 22–24]. Those cases are summarized in Table 1. Partial response was reported in one case only, with the combination of paclitaxel and carboplatin [22]. Other reports of target therapy treatment in this entity were not found.

**Table 1:** Summary of patients with epithelioid hemangioendothelioma treated with anti-angiogenic therapy

First author (ref)	No.pts.	Median age	Gender	Treatment medications	Response
Gaur S [6]	1	35	M	Bevacizumab, Nab-Paclitaxel	SD
Belmont [22]	1	41	M	Bevacizumab, Carboplatin, Paclitaxel	PR
Kim [15]	1	44	F	Bevacizumab, Carboplatin, Paclitaxel	PD
Lopes [16]	1	51	M	Bevacizumab, Carboplatin, Etoposide	PD
Mizota [23]	1	59	F	Bevacizumab, Carboplatin, Paclitaxel	PD
Ye [9]	1	44	F	Bevacizumab, Carboplatin, Paclitaxel	SD
Lazarus [24]	1	42	M	Bevacizumab, Paclitaxel	PD
	1	42	M	Carboplatin, Etoposide	PD
Salech [25]	1	40	F	Thalidomide	PR
Raphael *et al.* [26]	1	53	F	Thalidomide	SD
Kassam *et al.* [27]	1	13	F	Thalidomide	PD
Bolke *et al.* [28]	1	47	M	Thalidomide	PD
Mascarenhas *et al.* [29]	1	52	M	Thalidomide	PR
Pallotti *et al.* [31]	1	73	M	Lenalidomide	SD
Sumrall *et al.* [30]	1	31	F	Lenalidomide	SD
Agulnik *et al.* [18]	2	NA	NA	Bevacizumab	PR
	1	NA	NA	Bevacizumab	PD
	4	NA	NA	Bevacizumab	SD
Chevreau *et al.* [19]	5	NA	NA	Sorafenib	SD
	2	NA	NA	Sorafenib	PR
	8	NA	NA	Sorafenib	PD

*PR* partial response, *PD* progressive disease, *SD* stable disease

Although the mechanism of action of thalidomide and its analog, lenalidomide, is not fully understood, they are believed to have immunomodulatory as well as anti-angiogenic properties that logically can fit the treatment of this rare malignancy. A PubMed search using “thalidomide” and “hemangioendothelioma” identified five case reports [25–29], while “lenalidomide” and “hemangioendothelioma” identified only two case reports [30, 31]. However, none of these had primary thoracic involvement. These cases are also summarized in Table 1, which shows that two cases had partial responses that lasted up to 9 years in one case and another two patients had stable disease lasting up to 7 years.

## Conclusions

In conclusion, based on the presentation of VEGFR1in pulmonary epithelioid hemangioendothelioma cells, target therapies that block VEGFR have a logical base in this rare malignancy. The current case is the first to report objective, long-lasting response to pazopanib.

## Consent

Written informed consent was obtained from the patient for publication of this Case Report and any accompanying images. A copy of the written consent is available for review by the Editor of this journal.
